# Plasma Torquetenovirus (TTV) microRNAs and severity of COVID-19

**DOI:** 10.1186/s12985-022-01812-3

**Published:** 2022-05-13

**Authors:** Maria Alfreda Stincarelli, Andreina Baj, Bernardo Guidotti, Pietro Giorgio Spezia, Federica Novazzi, Ersilia Lucenteforte, Silvia Tillati, Daniele Focosi, Fabrizio Maggi, Simone Giannecchini

**Affiliations:** 1 Department of Experimental and Clinical Medicine, University of Florence, Viale Morgagni 48, 50134 Florence, Italy; 2 Department of Medicine and Surgery, University of Insubria, 21100 Varese, Italy; 3 Department of Translational Research, University of Pisa, 56100 Pisa, Italy; 4 Department of Clinical and Experimental Medicine, University of Pisa, 56100 Pisa, Italy; 5 North-Western Tuscany Blood Bank, Pisa University Hospital, 56124 Pisa, Italy

**Keywords:** *Anelloviridae*, Torquetenovirus, MicroRNA expression, SARS-CoV-2, COVID-19, Inflammatory response

## Abstract

**Background:**

Torquetenovirus (TTV), a widespread anellovirus recognized as the main component of the healthy human virome, displays viremia that is highly susceptible to variations in immune competence. TTV possesses microRNA (miRNA)-coding sequences that might be involved in viral immune evasion. Among TTV-encoded miRNAs, miRNA t1a, t3b, and tth8 have been found in biological fluids. Here, the presence of TTV DNA and TTV miRNAs in the plasma of severe acute respiratory syndrome coronavirus 2 (SARS-CoV-2)-infected subjects was investigated to monitor the possible association with coronavirus disease 2019 (COVID-19) severity.

**Methods:**

Detection of TTV DNA and miRNA t1a, t3b, and tth8 was investigated in plasma samples of 56 SARS-CoV-2-infected subjects with a spectrum of different COVID-19 outcomes. TTV DNA and TTV miRNAs were assessed with a universal single step real-time TaqMan PCR assay and miRNA quantitative RT-PCR miRNA assay, respectively.

**Results:**

The TTV DNA prevalence was 59%, whereas at least one TTV miRNA was found in 94% of the patients tested. miRNA tth8 was detected in 91% of subjects, followed by miRNAs t3b (64%) and miRNAt1a (30%). Remarkably, although TTV DNA was unrelated to COVID-19 severity, miRNA tth8 was significantly associated with the degree of disease (adjusted incidence rate ratio (IRR) 2.04, 95% CI 1.14–3.63, for the subjects in the high severity group compared to those in the low severity group).

**Conclusions:**

Our findings encourage further investigation to understand the potential role of TTV miRNAs in the different outcomes of COVID-19 at early and late stages.

## Introduction

Torquetenovirus (TTV) is the most representative virus of the *Anelloviridae* family. It is a small virus with a circular single-stranded DNA genome and represents the main component of the healthy human virome [1–3]. TTV was discovered in 1997 [4, 5] and consists of at least 29 genetically different species included in the genus Alphatorquevirus [2, 3, 6]. TTV remains highly prevalent after being acquired early in life, showing higher serum viral loads in immunosuppressed patients than in healthy patients and lacking definitive association with any human illness [7, 8]. TTV encodes microRNAs (miRNAs), small noncoding 22 nucleotide-long RNAs that are thought to play a role in evading the immune response and regulating viral reactivation and pathogenesis [9, 10]. Additionally, TTV miRNA expression was reported in different biological fluids (plasma, cerebrospinal fluid) of subjects infected with HIV, HCV, or HBV, transplant recipients, and healthy subjects with TTV DNA-positive and -negative viremia [11–13]. However, TTV miRNA expression and its relationship with host immunity remain undefined.

Since the introduction of severe acute respiratory syndrome coronavirus 2 (SARS-CoV-2) in humans in 2019, its pandemic transmission worldwide has become a great concern for public health [14, 15]. A poor disease outcome of coronavirus disease 2019 (COVID-19) has been associated with several risk factors (older age and chronic diseases). Although high levels of broad spectrum serum inflammatory mediators, a significant decrease in human leukocyte gene expression, and a dysregulated antiviral interferon response are recognized risk factors for severe COVID-19, the role of the human virome has been poorly investigated [16, 17]. In this context, investigation of the interactions between the SARS-CoV-2 cytokine storm regulating the inflammatory response and persistent viruses can reveal a multifaceted relationship. Recently, it has been reported that TTV DNA load increases with the onset of COVID-19 and is reduced after its resolution in kidney transplant recipients [18]. However, another investigation pointed out that although TTV DNA load might be useful for mortality risk assessment in COVID-19 patients, it is a poor surrogate marker of inflammation [19]. Additionally, a potential role of TTV in immune senescence and increasing the risk for mortality in elderly people has been described [12]. As suggested, TTV can impair the normal physiology of infected cells as well as uninfected cells [20]. Thus, viral miRNAs in circulation can be of interest for monitoring the status of viral expression and their association with certain multifactorial diseases. This study aimed to investigate TTV miRNA expression in the sera of patients infected with SARS-CoV-2 with different COVID-19 outcomes.

## Materials and methods

### Patients and samples

After internal review board approval (protocol number: 165/2020), 56 COVID-19 patients (defined as having a SARS-CoV-2-positive nasopharyngeal swab with real-time RT–PCR) attending the units of a COVID-19 hospital were enrolled in the study and donated plasma samples after providing informed consent. Clinical outcomes were registered according to the highest rank in the World Health Organization (WHO) eight-point ordinal scale of COVID-19 severity [21]: asymptomatic status (0), no limitation of activities (1), limitation of activities (2), hospitalized without oxygen therapy (3), oxygen by mask or nasal prongs (4), noninvasive ventilation or high flow oxygen (5), intubation and mechanical ventilation (6), ventilation with additional organ support (7), or death (8).

### TTV DNA quantification

TTV infection was assessed by extracting viral DNA from 200 µl of plasma samples using the QIAamp DNA Mini kit (QIAGEN, Chatsworth, CA) and determining the presence and load of the TTV genome using a single step universal TaqMan real-time PCR assay [22]. As described, the PCR target is a highly conserved region within the 5′ untranslated region (UTR) of the TTV genome, and the assay is, therefore, capable of detecting all TTV genotypes hitherto described.

### TTV miRNA RT-PCR quantification

Total RNA was isolated from 250 µl of plasma processed with an Exosomal RNA extraction kit (Norgen Biotek Corp., ON, Canada) following the manufacturer’s protocol. miRNA expression was analyzed and quantified with a commercial quantitative RT-PCR miRNA assay (Life Technologies, Foster City, CA) and using primers targeting the miRNA region previously described [11]. TTV miRNA t1a of genogroup 1, TTV miRNA t3b of genogroup 3, and TTV miRNA tth8 of genogroup 5 were selected according to predictive computational analysis [10] and because they were the most prevalent in plasma samples of previously studied patients [11]. Each reaction was carried out in triplicate with 10 ng RNA and included negative controls (no template) and 10^1^–10^6^ copies of positive control (synthetic oligonucleotide template). The lower limit of detection was 10 copies of TTV miRNA per ng of RNA. As assessed by several reactions performed in preliminary experiments and under various conditions, the assay proved specific and reproducible. Preliminarily, each TTV miRNA oligonucleotide standard was tested without amplification of an unrelated target. The interassay variation was found to be less than 0.5 Ct.

### Statistical analyses

To fulfill the study objectives, standard descriptive statistics and association analyses were carried out. The dependent variable (WHO score) was defined by the different levels of disease, with a score of 0 corresponding to an asymptomatic status and a score of 8 indicating death, and treated as a dichotomous variable (0 = 0–4; 1 = 5–8). In the descriptive analysis, we considered the following dichotomous variables: TTV DNA copies/ml, miRNA t1a and miRNA t3b copies/µg RNA (“Positive” > 10 and “Negative” ≤ 10). As TTV miRNA tth8 exhibited a low number of “Negative” results (only 5 samples < 10 copies/µg RNA) and a high fluctuation in the copy number, we decided to choose the median value of 3080 copies/µg RNA of all the samples as the variable of miRNA tth8. Thus, the variable of miRNA tth8 was classified as “Positive” if > 3080 and “Negative” if ≤ 3080, according to the median value of all 51 positive samples.

We considered the following covariates: patients’ demographics (age, sex) and clinical status (cardiovascular comorbidities and other previous infections).

Continuous variables were reported as the mean and standard deviation if they were normally distributed or as the median and interquartile range if they were not normally distributed. Normality was tested using the Shapiro–Wilk normality test. Categorical variables were reported as numbers and percentages.

In the descriptive analysis, medians were compared using the Kruskal–Wallis test; means were compared using Student’s t test, and proportions were compared using Fisher’s exact test.

To evaluate the association between the highest WHO score and the independent variables load of TTV DNA and miRNA TTV of genogroups 1, 3, or 5, we calculated unadjusted and age- and sex-adjusted IRRs and corresponding 95% CIs using a Poisson regression model with robust variance. A *p* value < 0.05 was considered statistically significant. Statistical analyses were conducted using R version 4.1.0.

## Results

### Demographic, clinical characteristics, and TTV status of study subjects

We included 56 subjects, 27 (48.2%) of whom reported a peak with a WHO score ≥ 5. We observed a higher percentage of opportunistic infections and deaths among the group with WHO scores ≥ 5 (74% vs. 21%, *p* value = 0.000, and 33% vs. 3%, *p* value = 0.010, respectively, Table 1).

**Table 1:** Distribution of WHO scores according to the demographic and clinical characteristics of the 56 subjects

	WHO score ≥ 5 (N = 27)	WHO score < 5 (N = 29)	*p* value
Sex			
Female, N (%)	9 (33)	17 (59)	0.104
Male, N (%)	18 (67)	12 (41)	
Age, years			
Mean ± SD	66 (12)	65 (19)	0.856
< 65, N (%)	13 (48)	15 (52)	0.074
65–75, N (%)	10 (37)	4 (14%	
> 75, N (%)	4 (15)	10 (34)	
Cardiovascular comorbidities			
No, N (%)	22 (85)	18 (72)	0.451
Yes, N (%)	4 (15)	7 (28)	
Opportunistic Infections			
No, N (%)	7 (26)	23 (79)	0.000
Yes, N (%)	20 (74)	6 (21)	
Death			
No, N (%)	18 (67)	28 (97)	0.010
Yes, N (%)	9 (33)	1 (3)	
D-dimero, median (IQR)	2734 (733–8647)	473 (205–2479)	0.275
PCR, median (IQR)	106 (27–209)	9 (4–68)	0.370
First COVID-19 test, median (IQR)	24.5 (18–27)	21.5 (15–30.5)	0.257

According to the WHO score, no statistically significant differences were observed between the groups in terms of sex (*p* value = 0.104), age (*p* value = 0.856), or cardiovascular comorbidities (*p* value = 0.451). The prevalence of TTV DNA in plasma was 59% (33/56), with at least one miRNA in the plasma of the great majority of individuals (54 subjects, 96%), and 21% of subjects harbored all 3 TTV miRNAs. The miRNA tth8 was detected in 91% of subjects, followed by miRNAs t3b (64%) and t1a (30%). The mean TTV DNA viral load was 1.6 × 10^6^ copies/ml, with a median of 10^5^ copies/ml. TTV miRNAs exhibited a mean of 63, 171, and 4780 copies/µg RNA for miRNAt1a, miRNA t3b, and miRNA tth8, respectively (median of 50, 135, and 3080 copies/µg of RNA for miRNA t1a, miRNA t3b, and miRNA tth8).

### Statistical analysis of TTV miRNA association with the highest WHO score of COVID-19 severity

**Fig. 1:**
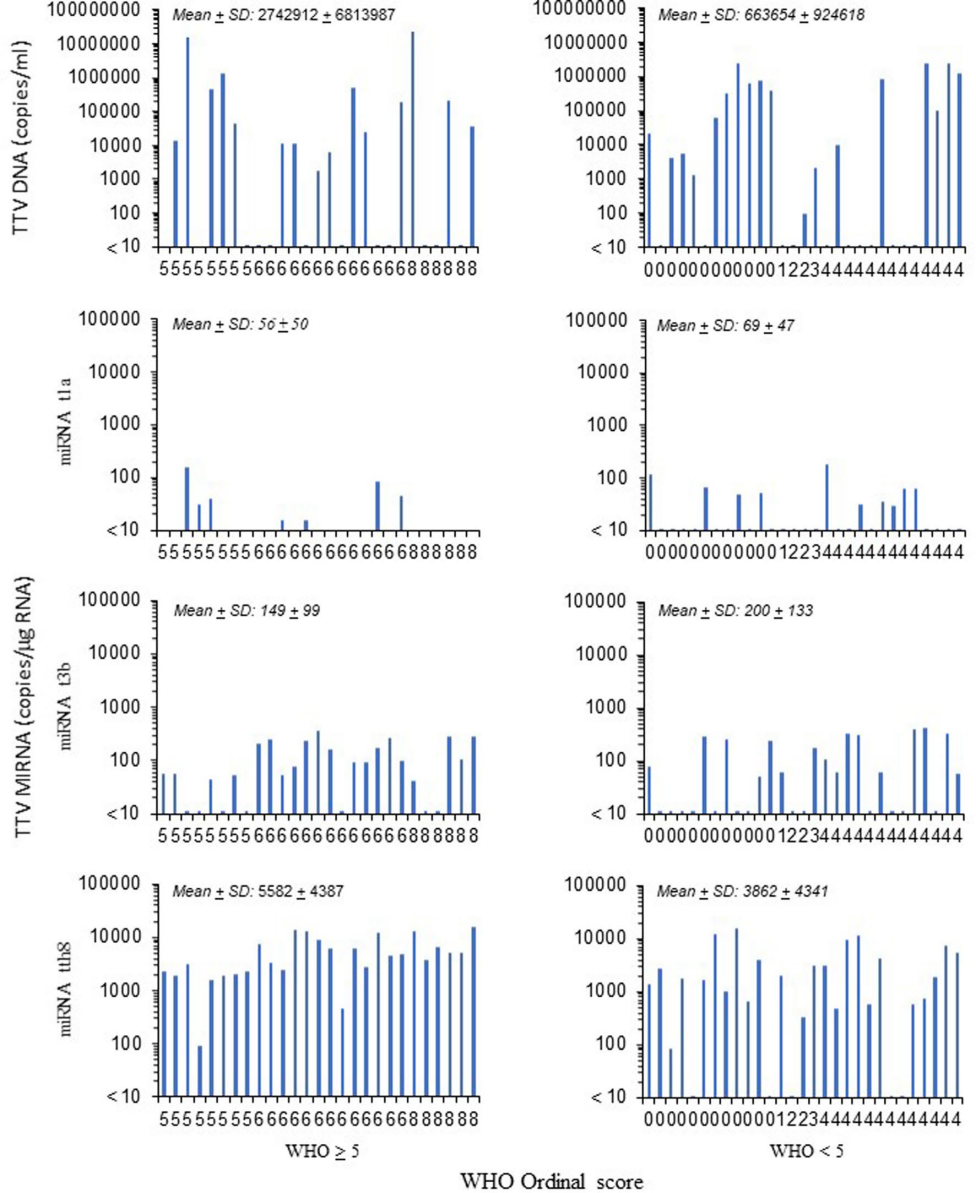
TTV DNA and miRNA quantification according to the WHO score of study subjects. Among the 27 samples with WHO ≥ 5, 15 (55%), 7 (26%), 20 (74%), and 27 (100%) were positive for TTV DNA, TTV miRNA t1a, TTV miRNA t3b, and TTV miRNA tth8, respectively. Among the 29 samples with WHO < 5, 18 (62%), 10 (34%), 16 (55%), and 24 (82%) were positive for TTV DNA, TTV miRNA t1a, TTV miRNA t3b, and TTV miRNA tth8, respectively. The mean ± standard deviation (SD) for the positive samples for TTV DNA, TTV miRNA t1a, TTV miRNA t3b, and TTV miRNA tth8 is also reported

Analyzing the TTV status according to the WHO score of COVID-19 severity, 15 out of 27 (55%) subjects with a WHO score ≥ 5 and 18 out of 29 (62%) subjects with a WHO score < 5 had plasma TTV DNA positivity (Fig. 1).

Moreover, 22 out of 27 (81%) subjects with a WHO score ≥ 5 and 16 out of 29 (55%) subjects with a WHO score < 5 had at least two TTV miRNAs. TTV DNA viral load exhibited a mean of 2.7 × 10^6^ and 6.6 × 10^5^ copies/ml (median of 44,880 and 208,114) in positive samples of subjects with a WHO score ≥ 5 and a WHO score < 5, respectively (Fig. 1).

The mean TTV miRNA copy number ranged from 56 to 5582 (median 39 to 4670) and from 69 to 3862 (median 56 to 1980) in subjects with a WHO score ≥ 5 and < 5, respectively, with the miRNA tth8 exhibiting the highest values. Table 2 shows that TTV DNA was not associated with COVID-19 severity. Conversely, when the three TTV miRNAs were investigated, miRNA tth8 was associated with COVID-19 severity (adjusted IRR 2.04, 95% CI 1.14–3.63, for the subjects in the high WHO score group compared to the low WHO score group, Table 2). Moreover, a direct association was observed between disease severity and miRNA t3b (adjusted IRR was 1.46, for the subjects in the high WHO score group compared to the low WHO score group), although the association was not statistically significant.

**Table 2:** Association between WHO score and MIRNA sequences. The results are reported as crude and sex- and age-adjusted incidence rate ratio (IRR) values and corresponding 95% confidence intervals (95% CIs)

	WHO score ≥ 5 (N = 27)	WHO score < 5 (N = 29)	IRR^a^ (95% CI)	Adjusted IRR (95% CI)
TTV DNA, copies/ml				
Negative (= 10), N (%)	12 (44)	11 (38)	Ref.	Ref.
Positive (> 10), N (%)	15 (56)	18 (62)	0.87 (0.51–1.50)	0.84 (0.47–1.51)
miRNA t1a, copies/ug RNA				
Negative (= 10), N (%)	20 (74)	19 (66)	Ref.	Ref.
Positive (> 10), N (%)	7 (26)	10 (34)	0.80 (0.42–1.53)	0.77 (0.41–1.44)
miRNA t3b, copies/ug RNA				
Negative (= 10), N (%)	7 (26)	13 (45)	Ref.	Ref.
Positive (> 10), N (%)	20 (74)	16 (55)	1.58 (0.82–3.09)	1.46 (0.74–2.88)
miRNA tth8, copies/ug RNA^b^				
≤ 3080, N (%)	10 (37)	20 (69)	Ref.	Ref.
> 3080, N (%)	17 (63)	9 (31)	1.96 (1.10–3.50)	2.04 (1.14–3.63)

^a^Incidence rate ratio (IRR) was calculated using Poisson regression models with robust variance

^b^As the TTV miRNA tth8 exhibited a low number of negative values, the median values (3080 copies/μg RNA) obtained using all positive data was used for dichotomous variables. Thus, for miRNA tth8, the data reported in the WHO score ≥ 5 and WHO score < 5 were the number of samples with values ≤ 3080 and those > 3080

## Discussion

In this study, the investigation of TTV status in the plasma of 56 subjects infected with SARS-CoV-2 developing different grades of COVID-19 severity had a prevalence of 59% of TTV DNA positivity, showing expression of at least one TTV miRNA in 94% of cases. The miRNA tth8 was detected in 91% of subjects, followed by miRNAs t3b (64%) and miRNA t1a (30%). These data confirmed that TTV miRNA expression can be present not only in TTV DNA-positive subjects but also in those where TTV DNA was not revealed [11, 13]. These results are consistent with the presence of TTV miRNA expression independent of TTV replication. Such a model has been previously proposed for other persistent viruses [23, 24]. Notably, although TTV DNA status was not associated with COVID-19 severity, miRNA tth8 was significantly associated.

COVID-19 has been associated not only with acute respiratory distress and pneumonia but also with chronic renal failure and myocardial inflammation [14, 15, 24, 25]. The most common symptoms have been attributed to the elevation of a broad spectrum of inflammatory mediators associated with poor disease outcomes. In this context, the direct or indirect viral interaction with transcription factors regulating the inflammatory response in COVID-19 is not completely understood. As demonstrated for other viruses, studies on SARS-CoV-2 have explored the modification of the miRNA processing pathway as a potential key player in the pathogenesis of COVID-19 [26, 27]. Thus, it cannot be ruled out that viral miRNAs encoded by persistent viruses to regulate their replicative cycle and escape immune response are also modulated, introducing additional cofactors in COVID-19 evolution. TTV miRNAs have been poorly investigated; although miRNA tth8 was initially associated with the regulation of the interferon pathway, such an association was not confirmed [10, 28]. In this context, persistent viruses constituting the human virome (such as herpesvirus and Polyomavirus) were shown to express viral miRNAs that control host physiology by targeting multiple processes, including the immune response [29]. Thus, viral modification of the miRNA processing pathway has been described as a potential key player in the regulation of the inflammatory response [26]. Several studies have suggested that there is an association between TTV miRNA expression and inflammatory status. For instance, it was reported that TTV miRNAs exhibit differential expression in healthy or diseased patients, with the highest values in transplant recipients [11, 13]. The miRNA t1a from TTV genogroup 1 was more prevalent in the plasma of diseased patients than of healthy donors (56% vs. 32%, respectively), whereas miRNAs of TTV genogroups 3 and 5 were not significantly different (87% and 51%, vs. 100% and 44%, for diseased and healthy subjects, respectively). Importantly, the overall prevalence of TTV genogroup 1 miRNAs was significantly higher in transplant recipients than in HIV- and HBV-infected patients [95% vs. 55% (11)]. Additionally, TTV miRNA t3b correlates with serum IL-6 levels, a marker of systemic inflammation, in elderly patients [12]. Of note, in the present study, TTV miRNA t3b status was associated with COVID-19 severity, although a small sample size prevented reaching statistical significance. Notably, the association of SARS-CoV-2 and TTV with the expression of human miR-181a (a regulator of cell proliferation, apoptosis, mitochondrial function, and immune response) has been investigated in patients with acute lymphoblastic leukemia [30, 31]. In that study, the expression of miR-181a was significantly increased in patients with COVID-19, whereas it was decreased in TTV-positive patients [31]. Moreover, the human virome in nasopharyngeal swab samples of SARS-CoV-2 patients has been recently investigated, showing TTV positivity in two subjects [32]. Thus, although it remains to be demonstrated, miRNA expression of a particular TTV genogroup present in the infected host might differentially modulate immune functionality, playing a role as an additional cofactor to the severity of the COVID-19 outcome.

Recently, oligoadenylate synthase-like (OASL) 1 protein, which is involved in the host's innate response against virus infection [33], has been identified as a risk factor for COVID-19 susceptibility and severity [34]. To date, TTV association with the inflammatory response has been reported for TTV ORF2 protein, which potentially interferes with the activity of NF-kB, a well-characterized transcription factor known to play a role in immune regulation [35]. Additionally, TTV DNA was associated with dose-dependent expression and production of proinflammatory cytokines by robust activation of TLR-9 in ex vivo grown mouse spleen cells [20].

Our study has several limitations, such as the small number of subjects enrolled. However, the relationship of TTV miRNA with the severity of the COVID-19 outcome should guide future studies investigating the role of the host virome at early and late time points after SARS-CoV-2 infection. This effort could be clinically relevant in the potential use of TTV miRNA expression to monitor host virome genetically acquired factor determining susceptibility to development of severe COVID-19 outcomes.

## Data Availability

The data presented in this study are available on request from the corresponding author.

## Abbreviations

TTV: Torquetenovirus
miRNA: MicroRNA
SARS-CoV-2: Severe acute respiratory syndrome coronavirus 2
COVID-19: Coronavirus disease 2019
WHO: World Health Organization
UTR: Untranslated region
IRR: Incidence rate ratios
OASL: Oligoadenylate synthase-like
